# 实体瘤免疫检查点阻断治疗中超进展性疾病的潜在机制研究进展

**DOI:** 10.3779/j.issn.1009-3419.2025.106.27

**Published:** 2025-09-20

**Authors:** Tingting LIU, Kai ZHU, Jiong DENG

**Affiliations:** ^1^256600 滨州，滨州医学院附属医院医学研究中心; ^1^Medical Research Center, Binzhou Medical University Hospital, Binzhou 256600, China; ^2^256600 滨州，滨州医学院附属医院核医学科; ^2^Department of Nuclear Medicine, Binzhou Medical University Hospital, Binzhou 256600, China

**Keywords:** 超进展性疾病, 免疫检查点阻断, 肿瘤微环境, 免疫逃逸, 代谢重编程, Hyper-progressive disease, Immune checkpoint blockade, Tumor microenvironment, Immune escape, Metabolic reprogramming

## Abstract

免疫检查点阻断（immune checkpoint blockade, ICB）疗法在多种癌症治疗中疗效显著，然而部分患者在接受此类治疗后却出现超进展性疾病（hyper-progressive disease, HPD），表现为肿瘤的加速生长及不良临床预后。本文对HPD的临床特征、潜在机制及可能的干预策略进行综述，以期为癌症临床治疗提供指导和建议。

## Abstract (en)

Immune checkpoint blockade (ICB) therapy has demonstrated significant efficacy in the treatment of various cancers. However, a subset of patients develops hyper-progressive disease (HPD) following ICB, which is characterized by accelerated tumor growth and poor clinical outcomes. This review outlines the clinical features, potential mechanisms, and possible intervention strategies of HPD, with the aim of informing clinical practice and providing relevant recommendations.

免疫检查点阻断（immune checkpoint blockade, ICB）疗法通过阻断程序性死亡受体1/程序性死亡配体1（programmed death-1/programmed death ligand 1, PD-1/PD-L1）或细胞毒性T淋巴细胞相关抗原4（cytototic T-lymphocyte-associated protein 4, CTLA-4）等免疫检查点分子，激活T细胞对肿瘤的免疫应答，已成为多种癌症的重要治疗手段。尽管ICB疗法在临床上疗效显著，但部分患者会出现超进展性疾病（hyper-progressive disease, HPD），表现为肿瘤迅速增大、疾病进展及患者生存期缩短^[1,2]^。HPD的发生机制尚不明确，但研究^[3]^表明，其可能与肿瘤内部因素如肿瘤基因组变化、宿主免疫系统的异常以及ICB治疗的免疫调节作用有关。在多种癌症，包括头颈部鳞癌（head and neck squamous cell carcinoma, HNSCC）、膀胱癌、三阴性乳腺癌、子宫内膜间质肉瘤、非小细胞肺癌（non-small cell lung cancer, NSCLC）和胃食管交界腺癌的肿瘤细胞中，鼠双微体蛋白基因2/4（murine double minute 2/4, *MDM2/4*）基因的扩增与HPD的发生密切相关，*MDM2*通过促进肿瘤细胞的增殖和抑制免疫细胞的活化来加剧病情^[4,5]^。此外，肿瘤微环境中不同类型免疫细胞的功能变化，如Foxp3^+^调节性T细胞（regulatory T cells, Treg）对效应T细胞的抑制，可能也是HPD发生的一个潜在机制^[6,7]^。在临床研究中HPD的发生率为4%-29%^[1,2]^。HPD不仅影响患者的生存期，还可能使后续治疗变得更加困难，很多患者可能因此错过其他治疗机会^[8,9]^。因此，识别HPD的潜在机制和生物标志物，对于改善患者的预后和优化治疗方案具有重要意义。

本文旨在系统综述HPD的潜在机制，探讨肿瘤内部因素、ICB治疗后固有免疫及适应性免疫变化等多方面的影响因素，为临床识别和干预HPD提供理论依据。

## 1 HPD的定义、临床特征及鉴别诊断

### 1.1 HPD的临床定义

不同研究对HPD的定义尚无统一标准，2016年的一篇个案报道^[10]^首次提出1例NSCLC患者在接受纳武利尤单抗治疗3个周期后原发病灶增大，双肺出现多个新发病灶并伴脑转移，称为“Disease Flare”。随着HPD报道的增多，通常用5个指标来界定HPD：（1）肿瘤生长速度比值（tumor growth rate ratio, TGR ratio）；（2）肿瘤生长速度绝对增量（ΔTGR）；（3）肿瘤生长动力学比值（tumor growth kinetic ratio, TGK ratio）；（4）实体瘤疗效评价标准（Response Evaluation Criteria in Solid Tumor, RECIST）；（5）治疗失败时间（time to treatment failure, TTF）。这5个指标中的一个或者几个指标的组合在不同的文献中被定义为HPD，具体信息见表1^[11-28]^。采用不同的定义，HPD发生率差异较大。TTF是唯一一种导致总生存期（overall survival, OS）显著恶化的定义^[29]^。由RECIST 1.1判定为疾病进展（progressive disease, PD）是大多数HPD发生的基础。PD发生后TGR比值≥2或者肿瘤增加超过10 mm或ΔTGR>50%都可确定HPD的发生。虽然HPD的定义随着研究的深入逐步完善，但仍存在一些问题，如HPD评价过程中至少需要2-3次影像学检查，很多患者由于缺乏足够的影像学资料可能被排除，影响临床治疗判断。同时，腹腔胸腔积液、淋巴结转移情况缺乏评价标准也可能低估HPD的发生率。因此，需要建立一种更加全面的共识性的HPD评价标准才能在临床上更好地评估和管理HPD的发生。

**表1:** HPD评价标准

Criteria of HPD	Tumor type	Treatment lines	Number of imaging evaluations	Reference
①PD; ②TGR ratio≥2	Different tumors	Anti-PD-1; Anti-PD-L1	3	Champiat, 2017^[11]^; Kanjanapan, 2019^[12]^; Tunali, 2019^[13]^; Sasaki, 2019^[14]^
①TTF<2 months; ②>50% increase in tumor burden compared to pre-immunotherapy imaging; ③>2-fold increase in progression pace	NSCLC; Melanoma; Gastric cancer	Anti-PD-1; Anti-PD-L1; Anti-CTLA-4	3	Kato, 2017^[15]^; Hagi, 2020^[16]^
TGK_R_≥2	HNSCC; Different tumors	Anti-PD-1; Anti-PD-L1	3	Saâda-Bouzid, 2017^[17]^; Karabajakian, 2020^[18]^; Park, 2020^[19]^; Economopoulou, 2021^[20]^; Long, 2023^[21]^
①PD; ②lesions increase ≥10 mm plus: a) increase of ≥40% in sum of target lesions compared to baseline; and/or b) increase of ≥20% in sum of target lesions compared to baseline plus the appearance of new lesions in at least 2 different organs	Different tumors	Dual blockade of TGF-β and PD-L1 pathways	2	Matos, 2020^[22]^; Chiang, 2023^[23]^
①PD; ②ΔTGR>50%	NSCLC	Anti-PD-1; Anti-PD-L1	3	Ferrara, 2018^[24]^
①PD; ②TGK_R_≥2; or TGR ratio≥2; or TTF<2 months	NSCLC	Anti-PD-1; Anti-PD-L1	3	Kim, 2019^[25]^
①PD; ②ΔTGR>100%	NSCLC	Anti-PD-1; Anti-PD-L1	3	Kas, 2020^[26]^
①TTF<2 months; ②TGK_R_≥2; ③>volume increase of 50% compared with baseline	NSCLC	Anti-PD-1; Anti-PD-L1	3	Kim, 2019^[27]^
3 out of the 5: ①TTF<2; ②>volume increase of 50% compared with baseline; ③2 new lesions; ④new organ lesion; ⑤ECOG performance status decrease	NSCLC	Anti-PD-1; Anti-PD-L1	3	Lo Russo, 2019^[28]^

HPD: hyper-progressive disease; PD: progressive disease; TTF: time to treatment failure; TGK: tumor growth kinetic; TGR: tumor growth rate; NSCLC: non-small cell lung cancer; ΔTGR: The tumor growth rate (TGR) before and during treatment and variation per month; TGK_R_: tumor growth kinetic ratio; HNSCC: head and neck squamous cell carcinoma; PD-1: programmed death-1; PD-L1: programmed death ligand 1; CTLA-4: cytotoxic T-lymphocyte associated protein 4; ECOG: Eastern Cooperative Oncology Group; TGF-β: transforming growth factor-β.

### 1.2 HPD的流行病学特征

HPD的发生率在不同类型的癌症中差异显著^[3,30]^。NSCLC患者中HPD的发生率较高，而在其他类型的肿瘤患者中则相对较低^[31,32]^。高龄、女性、体力状况、既往肿瘤区域接受过放疗、乳酸脱氢酶（lactate dehydrogenase, LDH）高、中性粒细胞与淋巴细胞比值高、肿瘤负荷及特定基因突变[如*MDM2/4*及表皮生长因子受体（epidermal growth factor receptor, *EGFR*）扩增等]与HPD风险增加相关^[8,33,34]^。另外，癌症肝转移和患者初始全身状况不良可能会增加HPD的发生风险^[33,35]^。HPD患者的预后通常显著不良，OS和无进展生存期（progression-free survival, PFS）都显著低于未发生HPD的患者群体，且他们更容易出现临床症状的恶化^[31,34,36]^。这些流行病学特征对于临床医生制定个体化治疗方案，尤其是选择免疫治疗的患者群体，具有重要的指导意义。

### 1.3 HPD与假性进展鉴别诊断

肿瘤假性进展是指在免疫治疗后影像学显示肿瘤出现短暂性增大而后变小的过程，该现象主要是由于肿瘤内部免疫细胞浸润，肿瘤细胞坏死水肿所致，在黑色素瘤、NSCLC和HNSCC免疫治疗后都有发生，发生率为2%-10%^[37-39]^。不同于HPD，假性进展通常患者预后良好。目前可以鉴别诊断的手段有：（1）计算机断层扫描（computed tomography, CT）影像组学^[40,41]^及正电子发射计算机断层显像（positron emission tomography/CT, PET/CT）^[42]^来反映肿瘤内部免疫浸润情况；（2）磁共振成像（magnetic resonance imaging, MRI）评估病灶内血流动力学^[43,44]^；（3）循环肿瘤DNA（circulating tumor DNA, ctDNA）^[45]^；（4）病理学检查^[46]^；（5）白介素-8（interleukin-8, IL-8）水平变化^[47]^。具体鉴别方法见表2。明确HPD与假性进展的鉴别标准，是后续精准解析HPD 发生机制、制定有效干预方案的基础，而肿瘤细胞自身的生物学特征改变，是探索HPD发生逻辑的首要维度。

**表2:** HPD与假性进展鉴别

Item	HPD	Pseudoprogression
Incidence rate	4%-29%	2%-10%
Prognosis	Poor	Good
Immune infiltration	Less	More
CT or PET/CT	Immune infiltration less	Immune infiltration more
MRI	Increased blood flow	Decreased blood flow
ctDNA	Increased	Decreased
Pathology	Cancer cell	Immune cell
Serum IL-8	Increased	Decreased

CT: computed tomography; PET: positron emission tomography; MRI: magnetic resonance imaging; ctDNA: circulating tumor DNA; IL-8: interleukin-8.

## 2 HPD的发生机制及预测指标

### 2.1 肿瘤细胞内在因素

#### 2.1.1 肿瘤细胞癌基因激活

Kato团队^[15]^在多变量分析中发现，NSCLC和黑色素瘤进行anti-PD-1/anti-PD-L1或anti-CTLA-4治疗后HPD（TTF<2个月）的发生与*MDM2/4*扩增和*EGFR*突变有关。随后，该团队进一步的研究^[48]^发现，1例胃食管结合部腺癌患者接受纳武利尤单抗治疗后发生HPD，该患者*MDM2/4*、人表皮生长因子受体3（Erb-B2 receptor tyrosine kinase 3, *ERBB3*）、丝氨酸/苏氨酸蛋白激酶家族基因（A-Raf proto-oncogene serine/threonine-protein kinase, *ARAF*）、细胞周期蛋白依赖性激酶4（Cyclin-dependent kinase 4, CDK4）和 *EGFR*基因扩增。*MDM2/4*介导HPD的发生可能有以下机制：（1）ICB治疗后肿瘤微环境中干扰素-γ（interferon-γ, IFN-γ）增加，激活肿瘤细胞JAK-STAT通路^[49]^，进而促进*MDM2/4*表达^[50]^。*MDM2/4*的主要功能是介导p53蛋白降解^[51]^，进而促进肿瘤生长及HPD的发生。（2）IFN-γ的增加可能通过干扰素调节因子8（interferon regulatory factor-8, IRF-8）促进*MDM2/4*表达^[50]^。（3）在肝癌免疫治疗超进展的研究中，含杆状病毒IAP重复序列蛋白5（baculoviral IAP repeat-containing protein 5, BIRC5）与*MDM2/4*相互作用促进HPD发生^[52]^。（4）*MDM2*作为E3泛素连接酶降解转录因子活化T-细胞核因子2（nuclear factor of activated T cells 2, NFATC2）从而降低T细胞活性^[53]^，诱导HPD发生。在一项黑色素瘤临床研究^[54]^中发现，*EGFR*突变和ICB治疗后HPD发生相关。*EGFR*可能通过诱导肿瘤细胞PD-L1表达驱动免疫逃逸进而促进HPD的发生^[55]^。另外，*EGFR*还可通过激活PI3K/AKT、STAT、RAS/RAF/MAPK和PLC/PKC通路促进肿瘤细胞增殖，这也是*EGFR*促进HPD发生的可能机制之一^[56]^。除了*MDM2/4*基因扩增及*EGFR*突变，HPD患者还存在胰岛素样生长因子1（insulin-like growth factor-1, IGF-1）、ERK/MAPK、PI3K/AKT和转化生长因子-β（transforming growth factor-β, TGF-β）上调^[57]^。*MDM2/4*和*EGFR*可能是预测HPD的分子标志物及治疗靶点。

#### 2.1.2 肿瘤细胞代谢通路改变

代谢重编程是肿瘤细胞为适应营养缺乏的肿瘤微环境而做出的生物能量代谢变化，是恶性肿瘤的一个显著特征，主要表现为糖酵解增强（Warburg effect）、脂质合成增加、异常的氨基酸及乳酸代谢^[58]^。Li团队^[59]^的研究表明，在NSCLC和黑色素瘤免疫检查点治疗后HPD的发生与糖酵解代谢有关。ICB治疗后CD8^+ ^T细胞活化分泌大量IFN-γ，IFN-γ通过抑制丙酮酸激酶活性抑制肿瘤细胞糖酵解及乳酸生成。具体的调节机制为T细胞分泌的IFN-γ促进肿瘤成纤维细胞生长因子2（fibroblast growth factor 2, FGF2）表达，从而抑制丙酮酸激酶同工酶2（pyruvate kinase type M2, PKM2）活性及降低烟酰胺腺嘌呤二核苷酸（nicotinamide adenine dinucleotide, NAD^+^）产生，造成β-连环蛋白（CTNNB1, β-catenin）通路激活重编程肿瘤干性，最终导致ICB治疗后HPD的发生。因此通过IFNg-PKM2-β-catenin通路的激活实现肿瘤免疫原性，代谢及致癌通路之间的相关串扰是ICB相关HPD发生的重要基石。靶向IFNg-PKM2-β-catenin通路可能是防止HPD发生的重要举措。另外在一项肝癌的研究^[60]^中发现，NAD^+^代谢同样影响肿瘤进展，通过靶向肝癌细胞中NAD^+^代谢驱动代谢网络之间相互作用可调节巨噬细胞极化模式从而介导免疫逃逸及HPD的发生。LDH是糖酵解最后一步的关键酶，能够催化丙酮酸转化为乳酸。在多种癌症中LDH在肿瘤细胞中表达上调并且与患者不良预后相关。通过抑制LDH减弱肿瘤细胞糖酵解过程可以抑制肿瘤生长和转移^[61]^。NSCLC临床研究表明，经ICB治疗后LDH水平升高与HPD的发生有关^[27]^，并且血清中LDH水平高于正常值2倍及以上与患者治疗后预后不良有关^[62]^。在治疗开始前LDH水平处于正常水平预后更好，但是治疗2个周期后LDH水平升高预后不良^[62]^，所以不能单靠基线期LDH水平评估患者ICB治疗反应，可动态监测血清LDH水平调整用药。

#### 2.1.3 肿瘤细胞内其他基因改变

ICB的重要靶蛋白PD-L1的表达也和治疗后HPD的发生相关。在NSCLC、HNSCC等肿瘤中进行anti-PD-1/anti-PD-L1治疗后外周血测序发现，PD-L1及血管内皮生长因子受体2（vascular endothelial growth factor receptor 2, VEGFR2）水平和HPD发生有关^[63]^。在一项胃癌的临床研究^[16]^中发现PD-L1低表达和HPD的发生有关，但是在该研究中仍有1例HPD患者基线PD-L1水平高达98%，这说明基线期PD-L1水平并不能直接决定患者是否可以在ICB治疗中获益，需要更大的样本量及研究进行确定。其他与HPD发生相关的基因改变，包括Kirsten大鼠肉瘤病毒癌基因同源物（Kirsten rat sarcoma viral oncogene homologue, *KRAS*）、丝氨酸/苏氨酸激酶11（serine/threonine kinase 11, *STK11*）^[27]^、单核细胞趋化蛋白1（monocyte chemoattractant protein 1, *MCP1*）、白血病抑制因子（leukemia inhibitory factor, *LIF*）、*CTLA-4*^[64]^、*RAD54L*^[65]^及Notch信号通路^[66]^，还未明确具体作用机制。

#### 2.1.4 肿瘤细胞干性增加

HPD的发生可能跟肿瘤细胞干性增加有关。文献^[67]^报道了1例66岁男性吸烟患者患有晚期肺腺癌，在经过帕博利珠单抗治疗后发生HPD，病理学检查显示肿瘤已转变为小细胞肺癌。可见，ICB之后使得肿瘤细胞去分化，干性增加，从而引起HPD。另外在小鼠乳腺癌模型^[68]^中发现，癌细胞与失活的CD8^+^ T细胞相互作用后增强的恶性潜能能够使BALB/cnu/nu小鼠的肿瘤生长和转移加剧，促使HPD发生。这表明无效的免疫反应不仅无法清除恶性肿瘤，还会激活癌细胞中促进干细胞特性及肿瘤播散能力的途径。肿瘤细胞的基因异常、代谢重编程及干性增强等内在改变，为HPD发生提供了核心“分子基础”，但肿瘤的免疫应答过程离不开微环境的调控，ICB治疗后肿瘤免疫微环境的动态重塑同样是驱动HPD的关键环节。

### 2.2 肿瘤免疫微环境变化

#### 2.2.1 固有免疫系统变化

有报道^[69]^表明PD-1抗体治疗后的小鼠对鼠沙门氏菌引起的急性肠道和全身感染更易感，这说明PD-1抗体治疗后损伤固有免疫系统，尤其是自然杀伤（natural killer, NK）细胞活性。有临床研究^[57]^发现，HPD患者3型天然淋巴细胞（group 3 innate lymphoid cell, ILC3）数量增加。ILC3是一种固有淋巴细胞亚群，可产生促肿瘤细胞因子IL-17、IL-22和粒细胞-巨噬细胞集落刺激因子（granulocyte-macrophage colony-stimulating factor, GM-CSF）^[70]^，这可能是ILC3引起HPD的一个原因。在卵巢癌的研究^[71-73]^中发现，肿瘤浸润的树突状细胞（dendritic cell, DC）高表达PD-1，发挥免疫抑制功能。ICB治疗后这些高表达PD-1的DC细胞分泌IL-10，而IL-10又促进DC细胞表达PD-1，如此形成恶性循环导致免疫抑制微环境，可能导致HPD发生。以上先天免疫系统在ICB后发挥的免疫抑制功能可能是造成HPD的原因，但是具体的分子调控机制还有待于进一步研究。

#### 2.2.2 适应性免疫系统变化

##### 2.2.2.1 细胞毒性T淋巴细胞功能异常

在肿瘤浸润的淋巴细胞中通常表达多种免疫抑制分子，包括PD-1、CTLA-4、T淋巴细胞免疫球蛋白黏蛋白3（T cell immunoglobulin and mucin domain-3, TIM3）和淋巴细胞活化基因3（lymphocyte activation gene-3, *LAG-3*）等。在卵巢癌的研究^[74]^中发现，单独使用PD-1、CTLA-4或者LAG-3抗体阻断并不能激活T细胞，反而使T细胞补偿性上调其他免疫抑制检查点而使T细胞处于耗竭状态，导致HPD的发生。小鼠实验中使用免疫检查点双重阻断或者三重阻断能够提高T细胞活性，降低肿瘤生成及生长速率。在HNSCC的肿瘤浸润T淋巴细胞进行PD-1阻断处理后，T细胞通过PI3K/AKT依赖而非细胞因子介导的途径提高TIM3表达，介导免疫逃逸及HPD的发生^[75]^。上述ICB后免疫检查点代偿性上调的发生使得T细胞处于更加耗竭的状态，增殖、迁移及细胞因子分泌功能严重障碍，是造成HPD发生的重要原因，因此检测外周血耗竭CD8^+^ T淋巴细胞可能预测HPD的发生。一项肺癌的临床研究^[76]^支持了这一说法，HPD患者外周血CD8^+^ T淋巴细胞效应/记忆亚群降低，严重耗竭细胞亚群（PD-1^+^TIGIT^+^CD8^+ ^T）增加。

##### 2.2.2.2 免疫抑制Treg细胞增殖

在晚期肺癌的临床研究^[77]^中发现，anti-PD-1治疗后HPD患者肿瘤浸润FoxP3^high^CD45RA^-^CD4^+ ^T细胞增加并且该细胞具有极强免疫抑制功能。并且在循环系统及肿瘤间质内，HPD患者Treg表达高水平PD-1和CTLA-4，从而发挥免疫抑制功能。因此，外周血Treg水平可能是ICB后HPD发生的一个预测指标^[78]^。同样在膀胱尿路上皮癌的研究^[79]^中发现ICB后HPD的发生与Treg细胞浸润增加有关。Treg细胞与效应T细胞在肿瘤中浸润失衡是HPD发生的一个重要原因^[80]^。一种针对Treg的抗体药物偶联物中引入近红外双卡霉素使其能够实现在肿瘤局部缓慢释放，既不破坏全身免疫系统又能清除肿瘤部位Treg细胞，提高ICB治疗效果^[81]^，这也可能是HPD治疗的一种可靠手段。

##### 2.2.2.3 肿瘤相关巨噬细胞浸润增加

一项肺癌的临床研究^[28]^发现，anti-PD-1治疗后HPD患者肿瘤浸润M2型巨噬细胞（CD163^+^CD33^+^PD-L1^-^）增多。Anti-PD-1抗体的Fc区域可以与巨噬细胞表面Fc受体相结合，诱导M1型巨噬细胞（抗肿瘤表型）向M2型（促肿瘤表型）转化，从而抑制抗肿瘤免疫反应，诱导HPD发生。肝癌免疫检查点抑制剂治疗后肿瘤浸润的CD103^+^细胞毒性T淋巴细胞可以与巨噬细胞相互作用，激活巨噬细胞NOD样受体热蛋白结构域相关蛋白3（NOD-like receptor pyrin domain containing 3, NLRP3）通路从而发挥免疫抑制功能，诱导HPD发生^[82]^。巨噬细胞在肿瘤微环境的作用下可转变为“HPD-related”表型，这种表型具有促炎表型并且兼顾有M1和M2特征^[83]^。

##### 2.2.2.4 骨髓源性抑制细胞浸润增加

ICB治疗后细胞毒性T淋巴细胞（cytotoxic T lymphocytes, CTLs）激活肿瘤细胞中NLRP3-热休克蛋白70（heat shock protein 70, HSP70）-Toll样受体4（Toll like receptor 4, TLR4）信号通路，从而招募髓系来源的抑制性细胞（myeloid-derived suppressor cells, MDSC）诱导HPD发生。对NLRP3或者HSP70进行基因或者药物抑制，能够阻止HPD的发生。基线血清中HSP70水平及肿瘤组织中NLRP3通路表达情况可能能够预测HPD的发生^[84]^。肿瘤细胞内在因素与免疫微环境的异常改变相互作用、形成调控网络，共同介导了ICB治疗相关HPD的发生，基于这些已明确的核心机制，探索针对性的干预策略成为改善患者预后的关键。

## 3 潜在干预策略

针对HPD的发生，联合治疗策略被认为是降低风险的有效方法。靶向治疗（如*MDM2*抑制剂）与ICB的结合可能会改善患者的预后并降低HPD的发生率。已有研究^[85]^表明，*MDM2*抑制剂能够通过激活p53通路，增强肿瘤微环境中抗肿瘤免疫反应，从而提高免疫检查点抑制剂的疗效。此外，联合使用靶向治疗与ICB能够通过调节免疫细胞成分和增强T细胞浸润来改善治疗效果，这为应对HPD提供了新的思路。例如，WEE1抑制剂的使用能够提高抗肿瘤免疫反应，并与PD-1抑制剂联合使用后显示出显著的抗肿瘤效果^[86]^。在一项针对晚期NSCLC患者的早期研究^[87]^中发现，纳武利尤单抗联合化疗应答率优于纳武利尤单抗单药治疗。CTLA-4抗体联合PD-L1/PD-1抗体可以降低HPD发生率^[88]^。在一项肠癌的研究^[89]^中发现，ICB联合代谢检查点药物能够提高治疗效果进而降低HPD发生率。优化治疗组合可降低HPD风险，未来的临床研究应重点探索各种联合治疗方案的安全性和有效性。上述基于HPD发生机制的联合治疗策略为降低HPD风险、提升ICB疗效提供了可行方向，结合当前研究现状，对HPD的核心机制与未来研究重点进行系统总结与展望，可为临床实践与科学探索提供更全面的指导。

## 4 总结和展望

HPD作为ICB治疗后的一种严重不良反应，其发生机制复杂且多样，涉及免疫检查点信号通路的异常、肿瘤微环境的变化、肿瘤的内在因素以及代谢重编程等多个方面。各核心机制间的相互作用及调控网络全貌通过图1进行凝练总结与可视化呈现。尽管当前对HPD的整体认识仍显不足，但通过对这些机制的深入研究，我们有望为未来的临床实践提供重要的指导，特别是在预测标志物的开发和干预策略的优化方面。

**图1:**
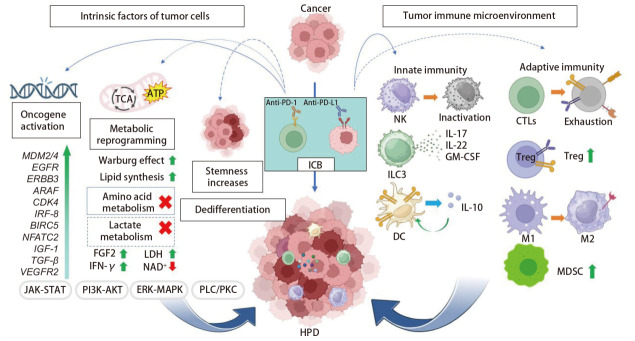
HPD发生机制图

展望未来，HPD研究的核心应围绕新型生物标志物的发现与验证展开。这不仅包括基因组学、转录组学和蛋白质组学等传统组学技术，还应积极探索单细胞测序、空间组学等前沿技术，深入挖掘肿瘤微环境的异质性及其与免疫治疗反应的关联。此外，治疗策略的优化同样关键。基于HPD机制的靶向治疗、新型免疫联合方案及个体化治疗设计有望显著改善HPD患者的临床结局。与此同时，加强临床试验设计的科学性和严谨性，提高患者筛选的精准度，也是提升治疗成功率的重要保障。
